# Water Detection in Urban Areas from GF-3

**DOI:** 10.3390/s18041299

**Published:** 2018-04-23

**Authors:** Xiaoyan Liu, Long Liu, Yun Shao, Quanhua Zhao, Qingjun Zhang, Linjiang Lou

**Affiliations:** 1 Laboratory of Target Microwave Properties, Deqing 313200, China; ltalexy@163.com (X.L.); shaoyun@radi.ac.cn (Y.S.); 2 Deqing Academy of Satellite Applications, Deqing 313200, China; 3 Laboratory of Radar Remote Sensing Application Technology, Institute of Remote Sensing and Digital Earth, Chinese Academy of Sciences, Beijing 100101, China; 4 Institute of Remote Sensing Science and Application, School of Geomatics, Liaoning Technical University, Fuxin 123000, China; zqhlby@163.com; 5 China Academy of Space Technology, Beijing Institute of Space System Engineering, Beijing 100086, China; ztzhangqj@163.com; 6 Satellite Surveying and Mapping Application Center, Beijing 100048, China; llj910808@hotmail.com

**Keywords:** GF-3 SAR images, building shadows, water detection, ROC curve

## Abstract

The rapid and accurate detection of urban water is critical for urban management, river detection, and flood disaster assessment. This study is devoted to detecting water by GaoFen-3 (GF-3) Synthetic Aperture Radar (SAR) images with high spatial resolution. There have been no effective solutions that discriminate water and building shadows using a single SAR image in previous research. Inspired by the principle that every shadow has a corresponding building nearby, a new method is proposed in this study, whereby building shadows are removed depending on the correspondence of buildings and their shadows. The proposed method is demonstrated effective and efficient by experimental results on six GF-3 SAR images. The Receiver Operating Characteristic (ROC) curves of the water detection results indicate that the proposed method increases the Probability of Detection (PD) to 98.36% and decreases the Probability of False Alarm (PFA) to 1.91% compared with the thresholding method, where, at the same PFA level, the maximum PD of the thresholding method is 72.62% in all testing samples. The proposed method is capable of removing building shadows and detecting water with high precision in urban areas, which presents the great potential of high-spatial-resolution GF-3 images in terms of water resource management.

## 1. Introduction

Urban water, an important factor in an urban ecosystem, is critical for improving urban development, the quality of the living environment, and the stability of the ecosystem [1,2]. In recent years, urban impervious surfaces have taken up greater proportions as urbanization has advanced, which has made urban floods frequent. Over 200 cities have seen flood disasters in varying degrees every year since 2000. Accordingly, urban traffic has been paralyzed, the lives and property of the people have been damaged, and buildings have been eroded. Accurate and timely water detection is crucial for real-time monitoring, effective prevention, and assessment of flood.

Remote sensing, with its advantages of spatial and temporal availability and manipulation of data covering large and inaccessible areas within a short time have become facilitate tools in the real-time monitoring of floods. Floods are often accompanied by cloudy and rainy weather conditions, which hampers the accessibility of optical images. Synthetic Aperture Radar (SAR), with the advantage of not being affected by terrible weather conditions, has become an irreplaceable data source for water information acquisition [3,4]. The Gaofen-3 (GF-3) satellite is China’s first 1 m spatial resolution C band multi-polarization SAR satellite. It is used for monitoring ocean environments and maintaining their rights and interests, monitoring and evaluating disasters, managing water resources, performing meteorological research, etc. This paper is written for water detection in urban areas and uses high-spatial-resolution GF-3 datasets.

Water detection is a classical topic in the scientific community. Most of the previous methods on water detection are based on image segmentation. Leng [5] used a Fuzzy C Means (FCM) algorithm to obtain three classes: water, background, and the intermediate area. The Nearest Neighbor Clustering (NNC) was then adopted as a second-level method to divide the pixels belonging to the intermediate area into water and background, respectively. Silveira [6] proposed a water/land separation approach using region-based level sets and adopted a mixture of log normal densities as a probabilistic model for pixel intensities in both water and land regions. Song [7] proposed a new active contour image segmentation algorithm inspired by cross entropy. Ciecholewski [8] proposed a river segmentation method that used watershed segmentation and combined regions by maximizing the average contrast. Yin [9] proposed an active contour model (ACM) with an edge indicator by assuming that the image boundary follows a Gibbs prior distribution.

The effectiveness of all the above-mentioned water detection methods based on image segmentation has been proved in many scenarios. However, building shadows is a critical distraction in water detection [10], especially in high-spatial-resolution SAR images, as both of these two ground objects present low backscattering behavior. In order to eliminate the impact of building shadows on water detection accuracy, several methods have been developed by introducing ancillary data or detailed a priori knowledge. Mason [11,12,13,14] used a SAR simulator in conjunction with high-resolution Light Detection And Ranging (LiDAR) data to estimate the shadow regions of the SAR image. Based on the imaging geometry of the SAR, these regions would not be visible due to the interaction of the radar beam and the building or vegetation in the urban area. Irwin [15] used the same method for surface water detection. Tanguy [10] successfully removed the building shadows by considering the flood Return Period (RP) data. In urban area, 87% of pixels were correctly identified, with under- and over-detection of 14%. Zhu [16] analyzed the radar observation parameters, house structure, and direction carefully with the help of detailed a priori knowledge, and proposed a decision rule to separate the shadow part from the water in SAR image. Pulvirenti [17] eliminated the shadow based on interferometric SAR (InSAR) data. The methods above are capable of achieving water detection by SAR images, but all of them require external ancillary data or detailed a priori knowledge, which increases the complexity of these methods in actual application.

In real scenes, people who obtain SAR images can easily discriminate building shadows from water. This discrimination is not only by the unique shape feature of building shadows, but also based on the obvious fact that every shadow corresponds with a building nearby. Inspired by such a basic principle, a new method is proposed by corresponding buildings with their shadows and by then using this correspondence to discriminate water regions from shadow regions. Since the correspondence is determined by searching along the tracing direction (the inversed shadow direction, from shadow to the corresponding building), the method is abbreviated as the TDS (Tracing Direction Searching) method.

The paper is mainly divided into two parts: Section 2 illustrates the methodology of the proposed method, including image segmentation and building shadows removal. Section 3 presents the results and discussion of the GF-3 SAR images, including the GF-3 datasets, the TDS method, and the comparison experiment. In this section, a validation of the effectiveness and efficiency of the proposed method and a comparison experiment with traditional thresholding method are presented.

## 2. Methodology

### 2.1. SAR Image Segmentation

Image segmentation is the basic step of applying classification rules in an object-oriented method. In order to obtain a mixture of water and building shadow, the paper adopts a fuzzy cluster segmentation algorithm that combines a Gaussian Mixture Model (GMM) [18] and a Hidden Markov Random Field model (HMRF) [19,20]. A brief introduction of this algorithm is presented in this section, while the details can be found in [21].

Given a SAR image $X=\left\{x_{j},j=1,\ldots ,n\right\}$, where *j* and *n* are the index and the number of pixels, respectively, $x_{j}$ is the intensity of pixel *j*. Assume that the image has *m* clusters, membership matrix $U={\left[u_{ij}\right]}_{n\times m}$ is adopted to represent image segmentation, where *i* is the index of cluster $u_{ij}\in \left[0,1\right]$, and $\sum_{j=1}^{m}u_{ij}=1$, $u_{ij}$ indicates the membership degree to which the *j*th pixel belongs to the *i*th cluster. By introducing KL regularization in the traditional Fuzzy C Means (FCM) algorithm, the objective function is redefined as

(1) $$\begin{array}{c} J=\sum_{j=1}^{n}\sum_{i=1}^{m}u_{ij}d_{ij}+\lambda \sum_{j=1}^{n}\sum_{i=1}^{m}u_{ij}\ln\left(\frac{u_{ij}}{w_{ij}}\right) \end{array}$$

where $d_{ij}$ denotes the non-similarity measure between the pixel and the clustering center, and $w_{ij}$ denotes the prior probability of the *j*th pixel belonging to cluster *i*th. λ is the fuzzy factor served for controlling the fuzzy degree of the algorithm with the value of 1<λ<∞. GMM is adopted to define the non-similarity measure for the statistical distribution in this paper. The formula of non-similarity measure $d_{ij}$ is

(2) $$\begin{array}{c} d_{ij}=-\ln w_{ij}p\left(x_{j}|v_{i},\Sigma_{i}\right)=\frac{q}{2}\ln\left(2\pi \right)+\frac{1}{2}\ln\left(|\Sigma_{i}|\right)+\frac{1}{2}{\left(x_{j}-v_{i}\right)}^{T}\Sigma_{i}^{-1}\left(x_{j}-v_{i}\right)-\ln w_{ij} \end{array}$$

where $v=\left\{v_{i},i=1,2,\ldots ,m\right\}$ is a set of clustering mean vectors, $\Sigma =\left\{\Sigma_{i},i=1,2,\ldots ,m\right\}$ is the set of covariance matrix, and $v_{i}$ and $\Sigma_{i}$ belong to the mean vector and covariance matrix of *i*.

The label field is defined as HMRF here. Let $L=(l_{1},l_{2},\ldots ,l_{n})$ be a label field of a given image *X* and $l_{j}\in \left\{1,2,\ldots ,m\right\}$ for the label of the *j*th pixel. The maximum membership degree criterion is adopted to achieve defuzzification, which determines the label of pixels:

(3) $$\begin{array}{c} l_{j}=\arg\max\left\{u_{ij};i=1,2,\ldots ,m\right\} \end{array}$$

where $l_{j}$ represents the label of pixel *j*. $j^{\prime }$ is the neighborhood pixel of the pixel *j*, and $l_{j^{\prime }}$ is the label of the neighborhood pixel. When the neighborhood pixels $j^{\prime }$ and the central pixels *j* have an identical label, a stable state is reached, and the potential energy $V_{m}\left(l_{j^{\prime }},l_{j}\right)$ is 0; otherwise, the potential energy $V_{m}\left(l_{j^{\prime }},l_{j}\right)$ is 1. The a priori probability of pixel *j* belonging to cluster *i* is

(4) $$\begin{array}{c} w_{ij}=\frac{\exp\left(-b\sum_{j^{\prime }\in N_{j}}V_{m}\left(l_{j^{\prime }},l_{j}\right)\right)}{\sum_{k=1}^{m}\exp\left(-b\sum_{j^{\prime }\in N_{j}}V_{m}\left(l_{j^{\prime }},l_{j}\right)\right)} \end{array}$$

where *b* represents the intensity parameter of the neighborhood labeling. A priori probability satisfies the condition $\sum_{i=1}^{m}w_{ij}=1$.

Finally, the membership degree $u_{ij}$, the converse derivation of the cluster center $v_{i}$, and the covariance matrix $\Sigma_{i}$ are expressed as follows:

(5) $$\begin{array}{c} u_{ij}=\frac{w_{ij}\exp\left(-\frac{d_{ij}}{\lambda }\right)}{\sum_{i^{\prime }=1}^{m}w_{ij}\exp\left(-\frac{d_{ij}}{\lambda }\right)} \end{array}$$

(6) $$\begin{array}{c} v_{i}=\frac{\sum_{j=1}^{n}x_{j}u_{ij}}{\sum_{j=1}^{n}u_{ij}} \end{array}$$

(7) $$\begin{array}{c} \Sigma_{i}=\frac{\sum_{j=1}^{n}u_{ij}\left(x_{j}-v_{i}\right){\left(x_{j}-v_{i}\right)}^{T}}{\sum_{j=1}^{n}u_{ij}}. \end{array}$$

**Figure 1:**
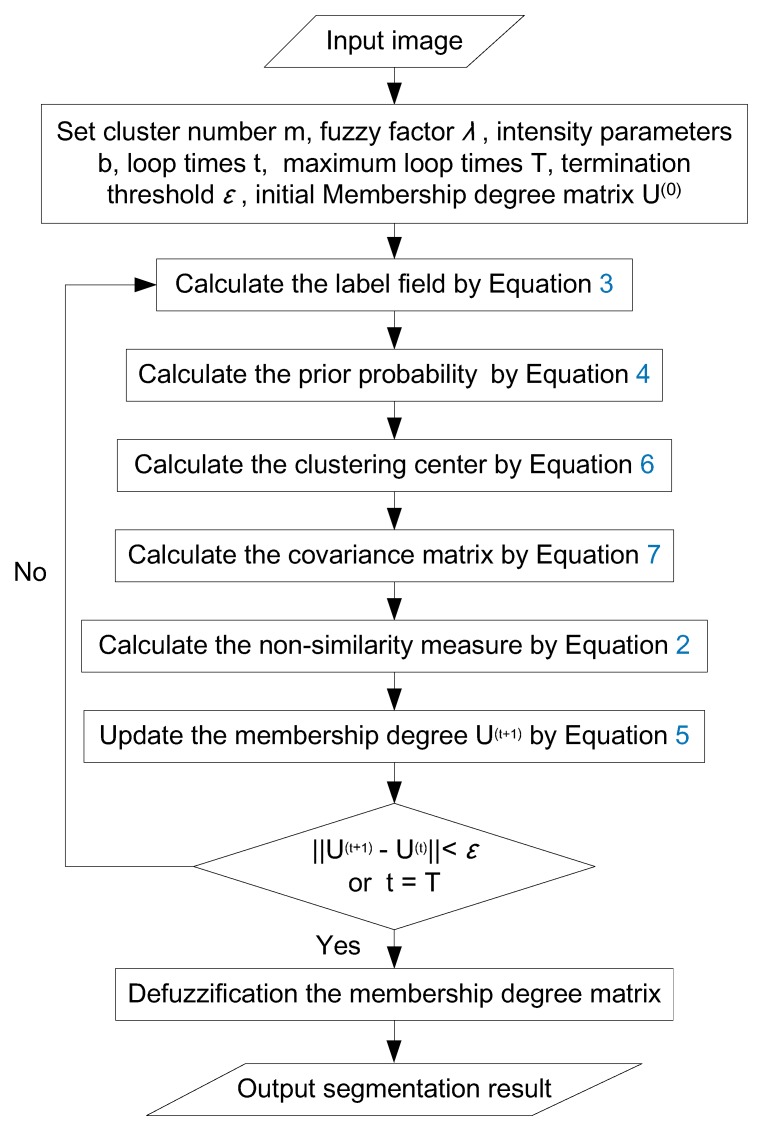
Graphical flowchart of the image segmentation method.

Figure 1 shows a graphical flowchart of the image segmentation method, and the essential parameters are presented in Table 1. In this paper, the SAR datum is classified into three clusters: (1) the building, (2) the fusion set of water, shadow, and several vegetation/bareland objects, and (3) all others. After sorting the three categories by the Digital Number (DN) value, the one with the lowest value is assigned as the fusion set, while the one with the highest value is assigned as the building. After segmentation results are obtained, to further eliminate the influence of noise, some minor patches (<*N* pixels, where *N* is determined by the demanded water areas, such as 50 $m^{2}$) are removed from the fusion set.

### 2.2. Building Shadows Removal—The TDS Method

Both the water and shadow objects are dark in the SAR images, although the scattering mechanisms behind them are different. A water body usually appears dark compared with the surrounding land because the smooth water surface acts as a specular reflector, while the shadow regions does not return radar signals to the relevant range bins. Usually, ancillary data or a priori knowledge is used to discriminate water and building shadows.

**Figure 2:**
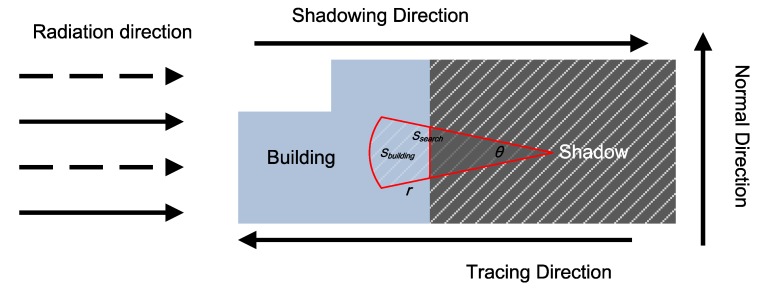
The principle of the shadowing direction.

The basic principle of the TDS method is that every shadow is near its corresponding building. Therefore, the corresponding building of the shadow can be determine along the tracing direction while water cannot. The radiation direction is incident direction of radar beam, and the vertical direction of the radiation is the normal direction; The shadow direction is from the building to the shadow, and the tracing direction is inverse. Figure 2 presents the correspondence between a building and its shadow. There are three main parts in the TDS method: the searching strategy, the definition of correspondence, and the rule to remove building shadow.

#### 2.2.1. Searching Strategy

Various searching strategies can be adopted along the tracing direction. In this paper, a fan-shape is utilized. By giving a narrow fan-beam, where the angle and radius of the fan-beam is θ and *r*, respectively, the building is expected to be searched. The starting point of this search is noted as $P_{\mathrm{center}}$, which is the mass center of the shadow region. θ is fixed $20^{\circ }$ based on the dimension of shadow objects along the normal direction. *r* is the maximum length between the boundary point $P_{\mathrm{boundary}}$ and the mass center of the shadow region, which could be represented as

(8) $$\begin{array}{c} r=\max_{P_{\mathrm{boundary}}\in C_{\mathrm{boundary}}}L\left(P_{\mathrm{boundary}},P_{\mathrm{center}}\right) \end{array}$$

where $C_{\mathrm{boundary}}$ is the boundary of the shadow region, and $L(P_{\mathrm{boundary}},P_{\mathrm{center}})$ is the Euclidean distance between $P_{\mathrm{boundary}}$ and $P_{\mathrm{center}}$.

#### 2.2.2. Definition of Correspondence

The correspondence between building and its shadow is measured by $P(S_{\mathrm{building}}\in S_{\mathrm{search}})$, which is defined as the geometry probability that the area of building belongs to the search area; $S_{\mathrm{search}}$ is the searching extent along the tracing direction. Specially, for the fan-shape search sector, $S_{\mathrm{search}}=\frac{1}{2}\theta r^{2}$; $S_{\mathrm{building}}$ is the area of the building located at the searching extent.

#### 2.2.3. The Rule of Shadow Removal

Based on the correspondence, the following rule is developed to remove the building shadow:

(9) $$\begin{array}{c} \mathrm{Shadow}:P(S_{\mathrm{building}}\in S_{\mathrm{search}})>\tau \\ \mathrm{Water}\;\mathrm{and}\;\mathrm{the}\;\mathrm{other}:P(S_{\mathrm{building}}\in S_{\mathrm{search}})\leq \tau \end{array}$$

where τ is a prespecified threshold value (τ=0.01 in this paper), which could be designed as self-adapted parameter in a future version. The value of τ is set predominantly based on the classification accuracy of the building. If the building extent could be extracted by the segmentation method accurately, τ could be set to zero, which means that, as long as the building is detected within the searching area, the correspondence between building and its shadow can be determined. However, in actual SAR images, the buildings are sometimes mixed with other man-made targets, such as street lamps and other metallic objects. Therefore, the threshold value is set to be higher than zero.

## 3. Results and Discussion

### 3.1. GF-3 Datasets

The GF-3 satellite, about 2779 kg in weight, was launched on 10 August 2016 by the China Academy of Space Technology (CAST). The orbital design life lasts for 8 years, and the orbit of the satellite is approximately 755 km. It carries a C-band SAR sensor with different polarizations, inclusive of single (HH or VV), dual (HH+HV or VH+VV), and full polarization (HH+HV+VH+VV). It covers 12 imaging modes with a spatial resolution of image ranging from 1 to 500 m and a swath coverage ranging from 10 to 650 km [22,23].

**Figure 3:**
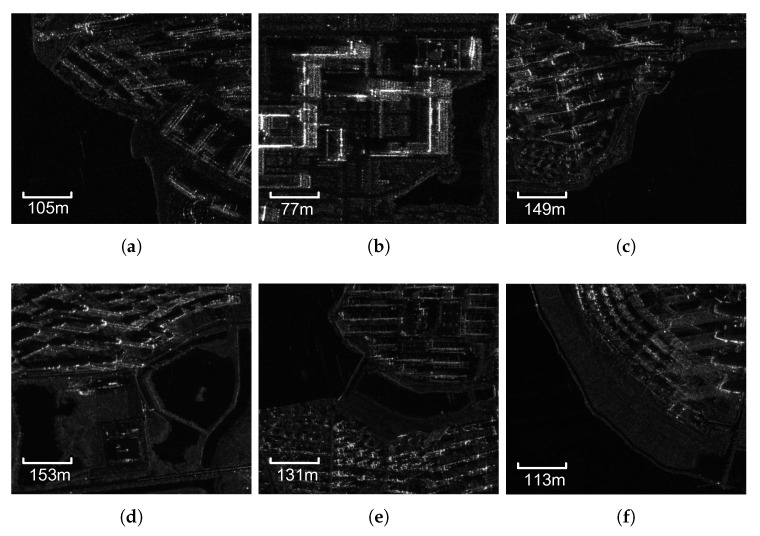
The original GF-3 image scenes. (**a**) HH; area: 0.25 km${}^{2}$. (**b**) VV; area: 0.14 km${}^{2}$. (**c**) HH; area: 0.50 km${}^{2}$. (**d**) VV; area: 0.53 km${}^{2}$. (**e**) HH; area: 0.39 km${}^{2}$. (**f**) HH; area: 0.29 km${}^{2}$.

To validate the universality of the proposed method, six SAR images are chosen by morphology differences, such as spatial resolution, coverage area, polarization mode (HH/VV), shadow direction, and building density. The water body also ranges from large open water to small regions, as shown in Figure 3 and the information of images are displayed in Table 2.

### 3.2. The TDS Method

**Figure 4:**
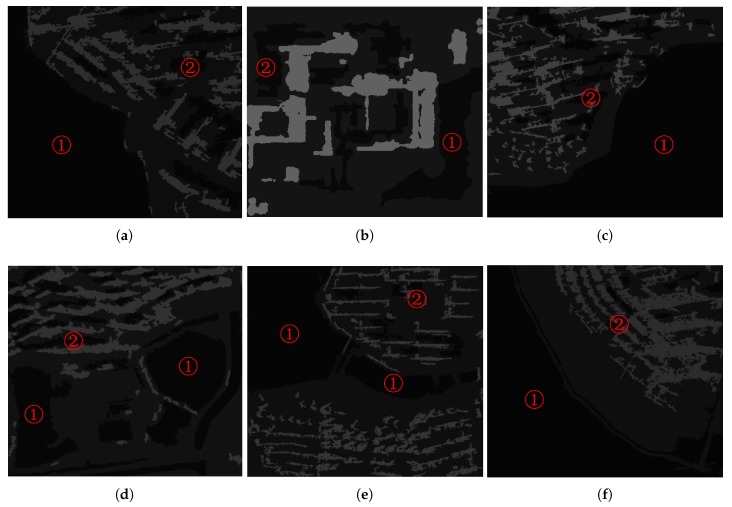
Segmentation results of GF-3 images (① represents water; ② represents building shadow).

**Figure 5:**
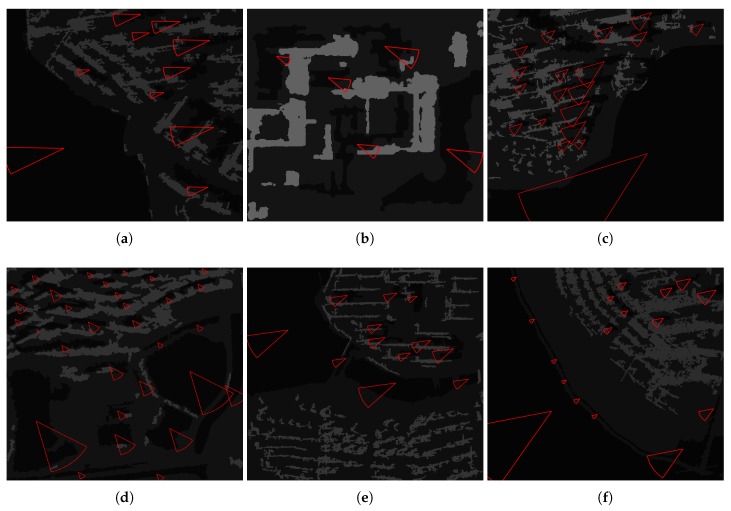
The fan-shape searching strategy from the shadow mass center to the corresponding building (the radius of the fan-shape searching sector is *r*, and the angle of the fan-shape is θ).

**Figure 6:**
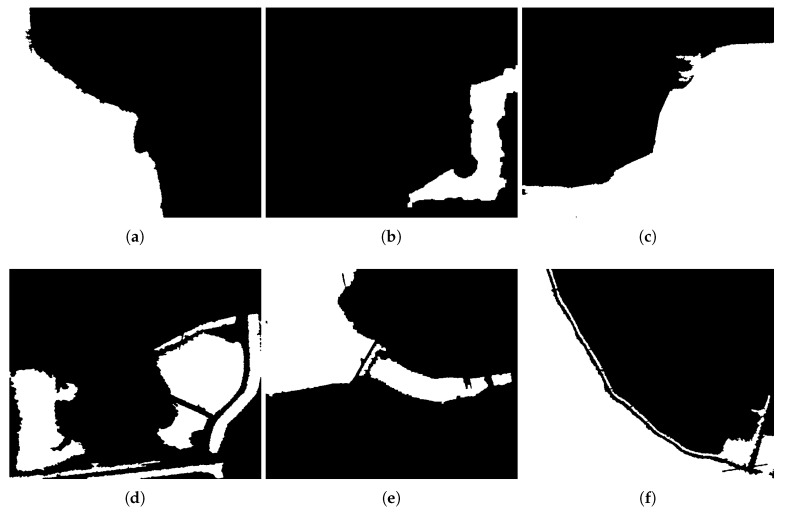
Water detection results of the Tracing Direction Searching (TDS) method.

**Figure 7:**
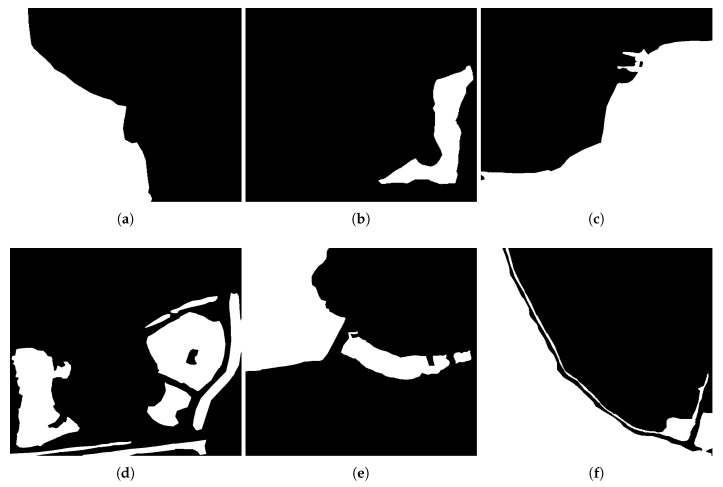
The ground truth data of GF-3 images by visual interpretation.

**Figure 8:**
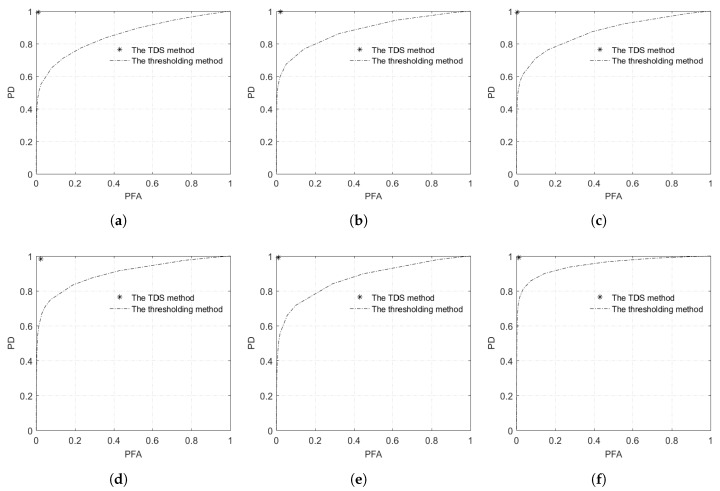
Receiver Operating Characteristic (ROC) curves of the TDS method and the thresholding method.

Figure 4 are segmentation results of the proposed method, where ① is an object of water, and ② is an object of building shadow. Figure 5 is a fan-shape searching strategy from the shadow mass center and its corresponding building, which perfectly reflects the basic principle. It is obviously that the searching fan-shape found none of the building from the mass center of ① along the tracing direction, while the building is successfully found from the mass center of ②. Figure 6 shows the water detection results. Figure 7 shows ground truth data of water extents, which are obtained by visual interpretation. The Probability of Detection (PD) and Probability of False Alarm (PFA) of the proposed method are 99.42%, 99.71%, 99.36%, 98.36%, 99.12%, 99.06%, and 0.86%, 1.58%, 0.33%, 1.91%, 0.93%, 1.11% respectively, as shown in Figure 8.

### 3.3. Comparison Experiment

To validate the effectiveness of the proposed method, a comparison experiment with the traditional thresholding method was conducted. Numerous methods, such as the OTSU [24,25] and Kittler–Illingnorth [26,27] methods, can obtain the optimum threshold value. In this paper, the threshold is attained by traversing all DN values in the image. The PD values of the thresholding method for the six GF-3 images, with the same PFA level of the proposed method, are 52.46%, 55.47%, 56.69%, 70.75%, 58.59%, and 72.62% respectively. ROC curves in Figure 8 show that the proposed method is vastly superior to the thresholding method.

**Figure 9:**
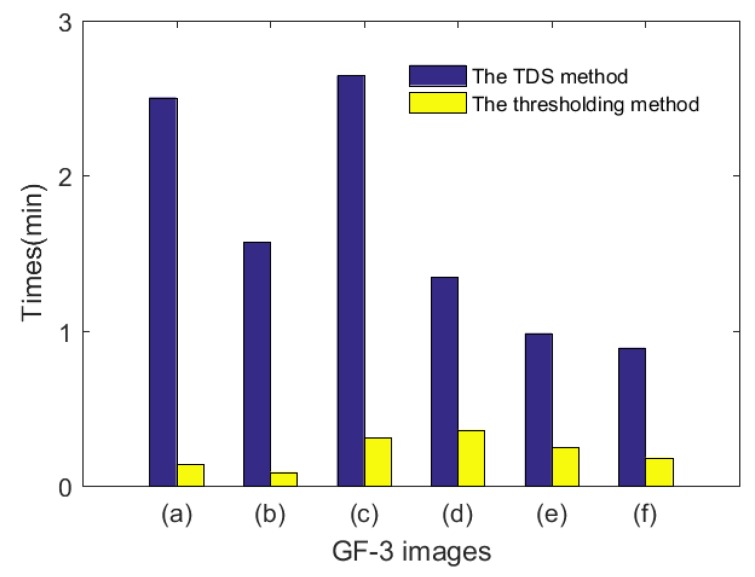
Computation efficiency of the TDS method and the thresholding method.

Both of these two methods, the TDS method and the thresholding method, were tested with the same computation environment: Intel Core2 2.53 GHz/4G Memory/MatlabR2016a. The computation efficiency comparison results of the two methods are shown in Figure 9. The TDS method is nearly 10 times slower than the thresholding method, which means that a greater computational cost is required to acquire higher water detection accuracy.

## 4. Conclusions

A novel water detection method based on GF-3 datasets is proposed in this study. The experimental results based on different polarization modes and image sizes indicate that the proposed method removes building shadows effectively. The ROC curves show that the proposed method greatly increases the PD and decreases the PFA compared with the thresholding method. The proposed method shows the great applicability and availability of GF-3 in detecting open water in urban areas.

The confusion between water and vegetation/bareland in the current validation experiments needs to be addressed. Numerous methods can identify vegetation/bareland with additional information, such as the Normalized Differential Vegetation Index (NDVI) or Soil Adjusted Vegetation Index (SAVI), with multi-spectral optical images, or with the help of multi-polarization modes. To extract water bodies with SAR images alone, the distinction between water and vegetation/bareland needs to be made. In addition, bad weather conditions and surface absorption (wet snow) will also enlarge the shadow regions [28] and therefore decrease detection accuracy. More research work is still required to achieve fully automated water detection algorithms that can perform, for example, a self-adapted determination of τ or can discriminate between water, low backscattering vegetation/bareland, dark regions caused by bad weather, and surfaces with absorption.

## Figures and Tables

**Table 1:** Parameters of the image segmentation method.

Cluster Number *m*	Fuzzy Factor λ	Intensity Parameter *b*	Loop Times *t*	Maximum Loop times *T*	Membership Degree Matrix $U^{\left(0\right)}$	Termination Threshold ϵ
3	0.2	0.3	0	100	randomly	0.01

**Table 2:** The information of GF-3 images.

No.	(a)	(b)	(c)	(d)	(e)	(f)
Time	2017.12.18	2017.01.02	2017.12.18	2017.01.02	2017.12.18	2017.12.18
Center Longitude (${}^{\circ }$)	114.37	114.50	114.35	114.41	114.38	114.29
Center Latitude (${}^{\circ }$)	30.50	30.49	30.50	30.45	30.47	30.45
Size (pixels)	504 × 490	387 × 347	756 × 659	725 × 724	647 × 600	551 × 525
Imaging Mode	SL	SL	SL	SL	SL	SL
Resolution (m)	1	1	1	1	1	1
Polarization	HH	VV	HH	VV	HH	HH
Coordinate	WGS-1984	WGS-1984	WGS-1984	WGS-1984	WGS-1984	WGS-1984
Incidence Angle (${}^{\circ }$)	40.60–41.23	40.36–40.96	40.60–41.23	40.36–40.96	40.60–41.23	40.60–41.23
Product Level	L2	L2	L2	L2	L2	L2
